# Supplementary material for: Calcium Affects Polyphosphate and Lipid Accumulation in Mucoromycota Fungi
Source: J Fungi (Basel). 2021 Apr 15;7(4):300. doi: 10.3390/jof7040300 (PMC8071181; doi:10.3390/jof7040300)
Supplement: Supplementary file 1 [file jof-07-00300-s001.zip › jof-1165989-SI.pdf]

Supplementary materials

Calcium affects polyphosphate and lipid accumulation in Mucoromycota fungi

Simona Dzurendova[1]*, Boris Zimmermann[1], Achim Kohler[1], Kasper Reitzel[2], Ulla Gro Nielsen[3], Benjamin Xavier Dupuy--Galet[1], Shaun Leivers[4], Svein Jarle Horn[4], Volha Shapaval[1]

[1]Norwegian University of Life Sciences, Faculty of Science and Technology, Drøbakveien 31, 1433 Ås, Norway

[2]Department of Biology, University of Southern Denmark, Campusvej 55, DK-5230, Odense M, Denmark

[3] Department of Physics, Chemistry and Pharmacy, University of Southern Denmark, Campusvej 55, DK-5230, Odense M, Denmark

[4]Norwegian University of Life Sciences, Faculty of Chemistry, Biotechnology and Food Science, Christian Magnus Falsens vei 1, 1433 Ås, Norway

* Correspondence: simona.dzurendova@gmail.com; simona.dzurendova@nmbu.no

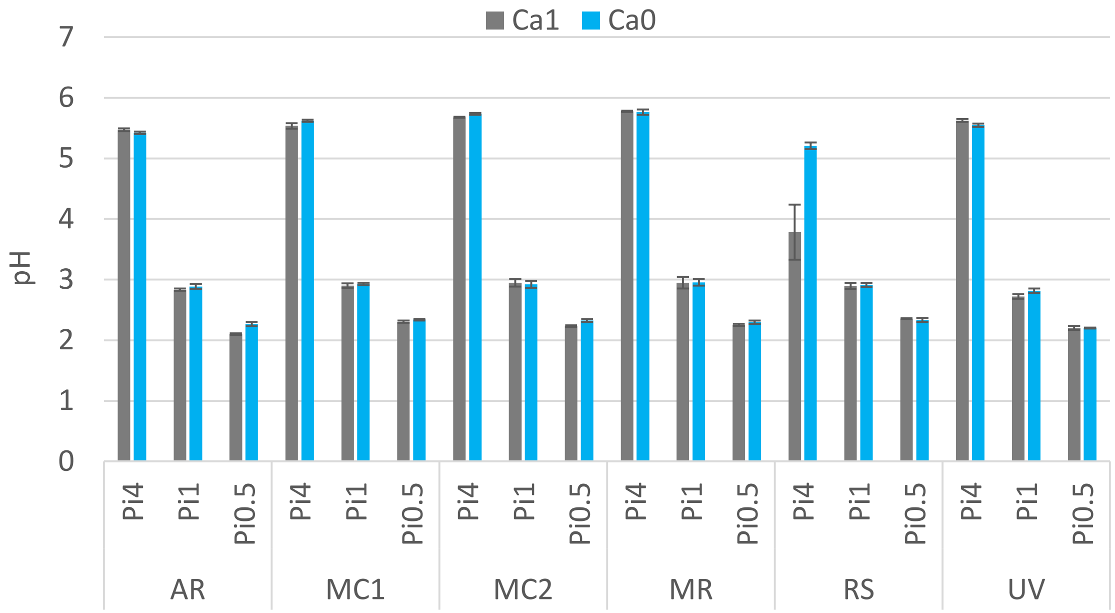

Figure S1: pH of culture supernatants at the end of the cultivation.

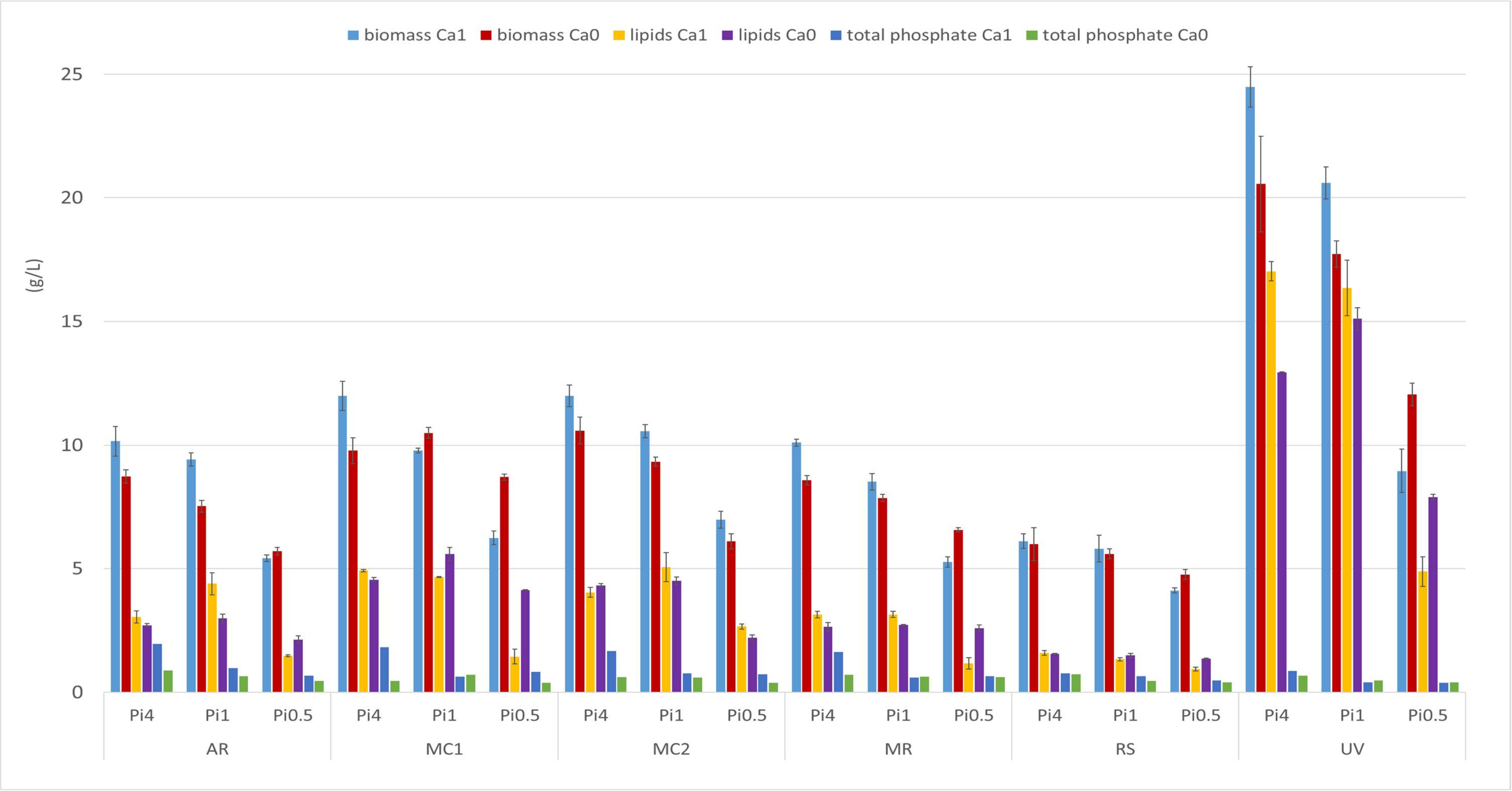

Figure S2: The total concentrations (in g/L) of fungal biomass, lipids and phosphates.

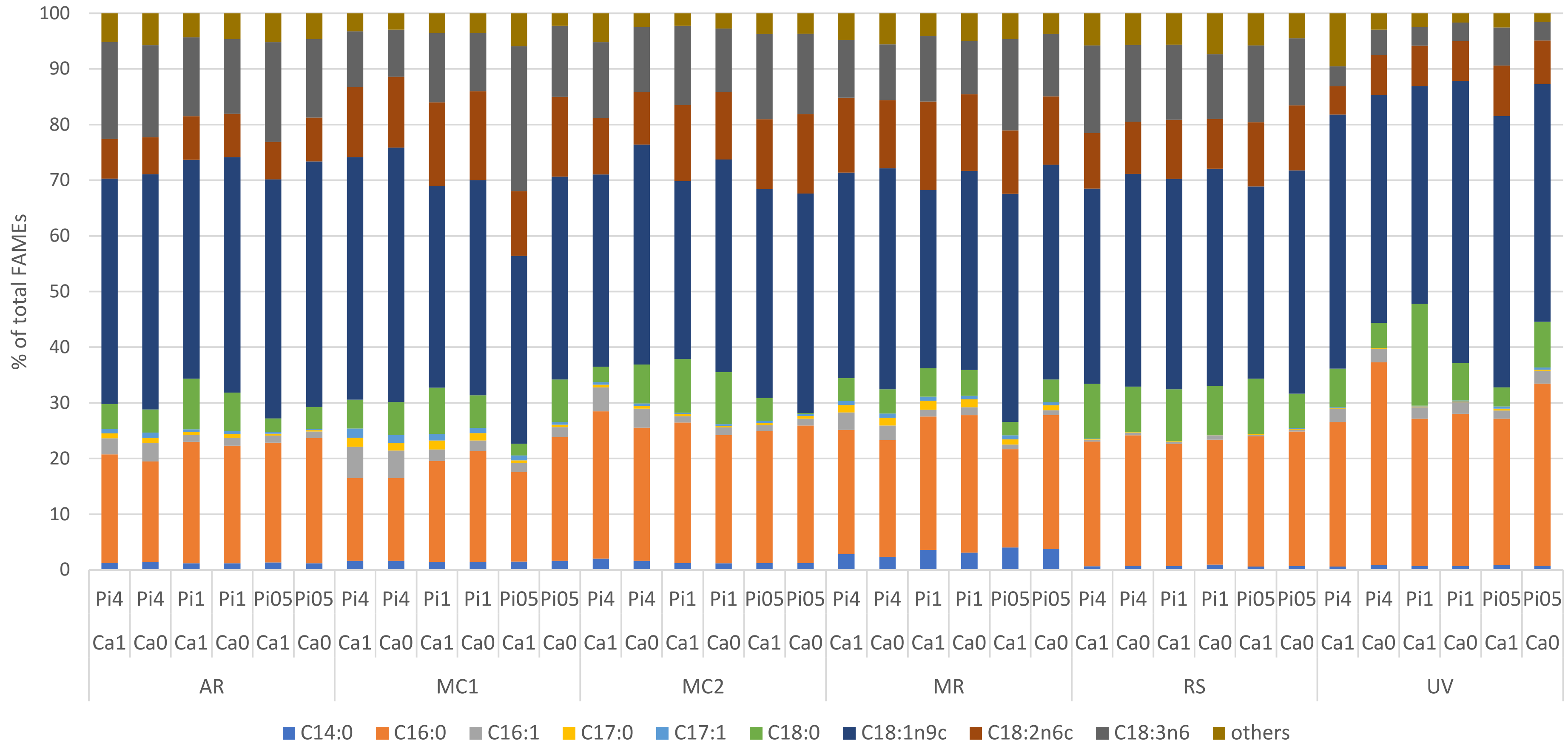

Figure S3: The fatty acid profiles of FAMEs extracted from fungal biomass. Fatty acids which were present in amount higher than 1% are shown, the rest is summed up into 'others'.
